# Stable Hygiene

**Published:** 1895-08

**Authors:** J. W. Sallade


### STABLE HYGIENE.[1]

BY J. W. SALLADE, V. S.

THIS may be said to be the science of the preservation of the health of our domestic animals. But it has a much wider significance, for upon the health of our animals often depends the health of our families and of ourselves.

The subject therefore concerns us not only in a monetary sense, in proportion to the amount of money invested in our live-stock, but the health of a community often depends upon the care and treatment given our dumb friends—the animals we employ in and about the farm, and whose produce enters so largely into food for the consumption of the human family.

When we consider that many of the most common and virulent diseases to which human flesh is heir are directly attributable to the consumption of these products, and that many of the ailments of domestic animals are directly and indirectly transmissible to the human family—in fact, have their origin there and are so transmitted—we can readily perceive why the utmost care and prudence should be exercised in the management of domestic animals, and why it is that strict sanitary laws should be enforced in the production of meat and milk. Large sums of money are annually expended by the government in investigating and stamping out contagious diseases affecting domesticated animals, and for a two-fold purpose, as already indicated. First, to prevent the great destruction of property, and, secondly, to maintain an equilibrium of health in

[1] Read before the Schuylkill Valley Veterinary Medical Association, June, 1895.

the human family. The labors of the Bureau of Animal Industry is most comprehensive and yet it is not sufficiently far-reaching to attain the desired results. It remains for bodies of this kind and kindred organizations, as well as individual effort, to carry on the great work and complete it. It is the province of the veterinarian and the medical practitioner to ferret out the causes and to provide the remedies for all abnormal conditions that give rise to disease; and it is the duty of the cattle-owner to practise strict hygiene, to exercise care in the production, preparation, and sale, so as to render his farm products wholesome instead of dangerous.

Stable hygiene embraces ventilation, drainage, disinfection, clean surfaces and surroundings, proper bedding, grooming, exercise, feed, water, etc. Too much stress cannot be laid on a proper attention to all these things.

Proper ventilation is too frequently overlooked, not only by farmers, but by owners of animals generally. The system generally in vogue is to close up all crevices, doors, windows (if any), and to labor under the mistaken idea that all pure air must be shut off in order to keep the stable warm. Animals are huddled together promiscuously in stables, the size of which is inadequate to furnish sufficient oxygen, and the result is that the animals are compelled to breathe over many times the same vitiated air, thus destroying all the life-giving principle and rendering the blood impure, and laying the foundation of numerous evils, among them being a condition of the system favorable for germs and microbes to propagate and pursue their deadly operations. A stable should be high and roomy, and provided with ventilators in the ceiling, or top ventilation, to carry off the obnoxious gases, such as carbonic acid gas and other effete and putrefactive matter. Where such ventilators are not provided, and the majority of barns are not so constructed, other means of admitting fresh air should be provided. A cold stable, even an open barn, is more healthful than an air-tight one, or one approaching that condition. In cases of pneumonia and other respiratory diseases the great mistake of closing up every crack (and thus shutting off that great remedial agent, oxygen) is too frequently made. In the lungs carbonic acid gas, a deadly agent, is given off and oxygen taken in to render the blood pure and arterial, and if already functionally impaired, unless pure air is supplied your case invariably proves fatal. Therefore the necessity of pure air in disease, and in order to maintain a healthy condition of the body the same rule must be observed. The object of hygiene is, as already stated, to preserve the health. A direct draught should be avoided and air admitted in such a way as to furnish an ample supply of pure atmosphere without subjecting the animal to a direct draught. In those cases proper clothing, bandages, etc., play an important part. Unless this condition is closely watched and regulated the original trouble will be followed by sequelæ or diseases more destructive than the original disease, such as blood-poison, purpura hemorrhagica, etc.

The stable should be built upon dry, high-lying ground, and provided with proper drainage. Great care should be exercised in the construction of stalls. They should be wide and spacious, sloping slightly from the manger toward the back, where a shallow gutter should be provided to catch and carry off the liquid excretions of the animal, and these gutters as well as the stalls should be kept scrupulously clean at all times. The surfaces (floor, wall, ceiling, etc.) should be kept clean. Cobwebs, etc., should be removed, mangers and hay-racks, if any, should be cleaned once a week, floors cleaned and swept, gutters, etc., flooded and washed, ceilings and walls swept and whitewashed at least twice a year and disinfected. The old practice in vogue on the farm of dunging out the stable but once in a week, is, to say the least, in opposition to good sanitation. The daily cleaning of the floor should be made a rule.

Pig-pens and chicken-coops require your attention. Do not permit them to take care of themselves. Clean them out; use plenty of lime in them; do not be afraid of the brush and broom. Keep the trough and vessels clean; provide plenty of pure fresh water. Scald and scrub your roosts, whitewash them often, remove the manure, provide sand, lime, charcoal, etc. Dust your floors and troughs in pig-pens with dry slacked lime; give your hogs once a week a liberal supply of lime water; provide comfortable places for these useful animals and keep them in proper condition. Clean liness is the first law in nature. If you want to achieve success in the hennery, and if you want to raise valuable pork, observe these rules. In order to get the best results from your chickens, house them warmly, give them a liberal supply of warm sloppy food at least once a day; supply them with meat, and, as already indicated, lime, sand, and other shell-producing ingredients. Labor is honorable.

Labor intelligently applied in and about the stable is remunerative. All these little items require your attention, and if properly observed and executed you will be astonished at the result.

Provide proper bedding for your larger animals. Shake it up daily. Do not permit it to lie in lumps and in an uneven manner. Do not have your horses standing on head or rest on an uneven bed at night; such a condition is not comfortable for the animal, and he will not be equal to the same amount of exertion as when comfortably bedded. The bed should be raised along the sides of the stall so that when the horse lies down his back is protected from the rough wall; as the sharp spinous processes of the vertebra, or spinal column, is not covered by anything but skin, considerable pain is caused when pressed against the hard wood or stone. In the morning the wet and dirty parts should be forked out and dry bedding pressed tightly under the manger or piled in a corner to give the floor a chance to dry. At night it should be spread evenly over the floor and covered with new straw. Sawdust, sand, refuse, peat, moss, and other materials are used for bedding. Whatever the material that may be used, all the dropping should be daily removed and the bed kept even, dry, and comfortable.

By the term dressing or grooming is generally understood the cleansing of the skin which the horse requires. He is never in the highest health unless the pores are kept free from scurf which forms on them whenever he sweats, and the object of the currying and brushing which he receives at the hands of his groom is to get rid of this mechanical obstruction as well as to brace the nerves of the surface by the friction of the brush and whisk. This dressing must be repeated daily, even if the horse has not been sweated. The only way by which this object is attained is to lay on the brush, whisk, etc., sharply. Use elbow-grease. It is not sufficient merely to remove the mud. When a horse is turned out to grass his coat often looks rough, yet his skin is washed by every rain and braced up by every wind, and he is cleaned by the waters of heaven. This is not so when kept indoors. The horse requires this care as much as you do your daily bath.

Nor is the horse the only one of your domestic animals that needs this care. You should devote some of the same care to your dairy-stock. Cows are too frequently neglected in this respect. In order that they may serve their purpose well they should receive especial care as regards cleanliness. Their bodies should be kept clean; all of their surroundings should be of an order calculated to make them comfortable. That they may yield pure and wholesome milk they should be kept in good health at all times, and unless they are so kept they become a source of the greatest danger. If I were to continue and enumerate all the diseases the cow is subject to, and tell you that consumption, or tuberculosis, and many other loathsome diseases are primarily found in the cow and are transmitted to the human family through milk and meat, you could readily understand the force of my assertion. But that is not the object of this paper. Suffice it to serve the purpose of this effort to state merely that such is the case, and that very frequently diseases, such as typhoid fever, for example, are traced to the dairy, and not always, if ever, due directly to the milk as it comes from the cow, yet nevertheless so conveyed. Milk is one of the very best mediums for the propagation of microbes, and the careless handling of this article of food, as already stated, is a prolific source of contamination. The udders of cows should engage your especial attention. If at all dirty they should be washed before milking. Your milk-pails and milk-cans should be clean and free from all putrefactive matter.

The water you use in and about your dairy shoul be pure. A case in point will better illustrate this argument. Several weeks since I was called to make a professional visit to a dairy located at the fever-stricken Mt. Carbon. Mr. Goulden, the owner, told me that people had stopped taking his milk, blaming it as the cause of the epidemic. His own family had the fever. I made a thorough examination of the cows and pronounced them healthy. I could find no symptom of disease in any of them, and yet every family that had the disease had been using the Goulden milk. The attending physician had investigated the cause and laid it at the door of a well from which the Gouldens used the water. I, too, inquired into the probable causes of the epidemic and came to the conclusion that the primary trouble laid in the extreme unsanitary condition of the place in general and in the drinking-water particularly. Along the lower end of the town, where the disease first appeared, there is the worst kind of drainage, if any at all. The river-bed is filled with culm and refuse matter from the slaughter-houses and sewerage of the town of Pottsville. The water-supply for household and stable purposes is obtained from wells in close proximity to the river, and only last summer the place was flooded. The pump-beds are low and in bad condition, and there is no doubt that the water in the wells was polluted, and that the flood deposited putrefactive matter about the place which gave rise to obnoxious and deadly effluvia, causing an epidemic of a horrible and death-dealing character.

Now, what has this to do with stable hygiene? Let me tell you. The milk as it came from the cows of the Goulden dairy contained no germs of disease, but later it may have carried the germs, and in large numbers. As already stated, milk affords a favorable field for the growth and multiplication of germs, and the fact that water was used to wash the pails and milk-cans (I would not say to dilute the milk, for I consider Mr. Goulden a very honest and upright citizen) is sufficient to have conveyed germs of the disease to the milk, where they increased in numbers and went forth to do their deadly work.

This shows the importance of pure water; and how often do we find cattle supplied from wells located in barnyards polluted by decomposed matter! This should not be so. Pay more attention to the kind of water you use in and about your stable and dairy.

So also with food. If possible, feed nothing but the best. Musty hay, oats, corn-fodder, or any other kind of musty feed is not fit for food and should not be used. Be careful in selecting, storing, and curing your winter-supply. Many diseases in the horse and other of our farm animals are due to these causes. Cerebro-spinal meningitis in the horse, a very fatal disease, is due to a microbe found in such kind of food. The feeding should be regular and sufficient in quantity to supply waste of the tissue, which is continually taking place, but more so when at exercise, violent or otherwise, and the animal in question must therefore be treated in this respect according to circumstances. One thing I wish to impress strongly, and that is watering before feeding, and never right after. The stomach of the horse is very small in proportion to the size of the animal. It only holds from three to three-and-a-half gallons. In feeding the horse the food should be taken in the following order: First, water; second, hay; third, oats; as the water, if given last, would wash the food into the intestines before it was acted upon by the gastric juice, while if hay was given after oats it would carry them along with it, as it is principally digested in the intestines, the oats being acted upon by the stomach for the most part.

The dehorning of cattle might be properly considered in this connection. In their wild state cattle need their horns and use them as weapons of defense; but in a state of domestication they abuse each other, gore and tear not only one another, but often attack other animals and even attendants. By removing them the cows become less vicious, will lie down together like a flock of sheep; a number will drink at the same trough; they will keep in better condition for producing milk; you can turn the cattle, horses, sheep, pigs, etc., all in the same barnyard; they require less room and can be handled with more safety. Dehorning diminishes the number of abortions, prevents tortures and losses, and protects yourself, your family, and your stock from injury and fear. Besides all this, it largely increases the butter-capacity of the cow, promotes peace and composure among them, which may account for this increase of butter-fats. The operation is not dangerous, and occupies but a few moments' time, if performed by a competent person. I would advise you to have your cattle dehorned, but have it done by one who understands the anatomy, and is therefore better fitted to operate successfully.

I alluded to disinfectants, and we can never dispense with them. They are purifying agents. They either neutralize or destroy the poisonous matter. We should use them frequently about the stable, in the dairy, and particularly all milk-pans, buckets, and milk-cans should be rinsed out with a solution of some one or other of these remedies. For dairy use I would recommend a weak solution of permanganate of potash; while for stall and stable use I know of none better, considering its cost, than a solution of sulphate of iron, or copperas. A half-pound of this, costing two cents, dissolved in a bucket of cold water and sprinkled all over the floor with a sprinkling-can, is sufficient to purify a whole stable, if first properly cleaned out.

In order to do this subject justice, I purpose to devote some of my leizure time to writing a small pamphlet or book, taking up one subject after another, treating it step by step, as best as I understand its importance. I shall also write a second part to it, treating of all the ordinary diseases of farm animals, describing them in common, every-day language (so that the layman may understand it thoroughly), and advise the best treatment that can be employed safely by the unprofessional.

In the meantime I advise to practise and observe the measures suggested in this humble effort, and if you do I feel confident that good results will be your reward, and that your dumb animals will appreciate your kindness. "The merciful man is merciful to his beast."

